# Investigation of the Wear Resistance of Hard Anodic Al_2_O_3_/IF-WS_2_ Coatings Deposited on Aluminium Alloys

**DOI:** 10.3390/ma18153471

**Published:** 2025-07-24

**Authors:** Joanna Korzekwa, Adam Jarząbek, Marek Bara, Mateusz Niedźwiedź, Krzysztof Cwynar, Dariusz Oleszak

**Affiliations:** 1 Institute of Materials Engineering, Faculty of Science and Technology, University of Silesia in Katowice, 75 Pułku Piechoty 1a, 41-500 Chorzów, Polandmarek.bara@us.edu.pl (M.B.); mateusz.niedzwiedz@us.edu.pl (M.N.); 2 Institute of Chemistry, Faculty of Science and Technology, University of Silesia in Katowice, Szkolna 9, 40-006 Katowice, Poland; krzysztof.cwynar@us.edu.pl; 3 Faculty of Materials Science and Engineering, Warsaw University of Technology, 02-507 Warsaw, Poland

**Keywords:** AAO coatings, IF-WS_2_ nanolubricant, wear resistance

## Abstract

The anodic oxide layer’s porosity is considered a functional feature, acting as a reservoir of lubricants. This feature enables the design of self-lubricating systems that effectively reduce friction and wear. To improve the tribological performance of Al_2_O_3_ anodic coatings on EN AW 5251 aluminium alloys, this paper presents a modification of the coating with tungsten disulfide (IF-WS_2_) nanopowder and its effect on coating resistance. The wear properties of Al_2_O_3_/IF-WS_2_ coatings in contact with a cast iron pin were investigated. The results include the analysis of the friction coefficient in the reciprocating motion without oil lubrication at two loads, the analysis of the wear intensity of the cast iron pin, the characterisation of wear scars, and the analysis of SGP parameters. Two-level factorial analysis showed that load and nanomodification significantly affected the load-bearing parameter Rk. Incorporation of the modifier, especially under higher loads, reduced the Rk value, thus improving the tribological durability of the contact pair. Both load and nanomodification had a notable impact on the coefficient of friction. The use of IF-WS_2_-modified coatings reduced the coefficient, and higher loads further enhanced this effect, by approximately 9% at a load of 0.3 MPa and 15% at a load of 0.6 MPa, indicating improved lubricating conditions under greater contact stress.

## 1. Introduction

High-strength aluminium alloys are widely employed in structural and mechanical applications where low weight and high mechanical performance are critical, notably in the automotive, aerospace, and machinery sectors [1,2]. Their favourable strength-to-weight ratio and corrosion resistance make them attractive for components subjected to dynamic loading and environmental exposure. However, under conditions involving sliding contact, aluminium alloys are inherently prone to adhesive wear and galling due to their relatively low surface hardness and high chemical reactivity. This limitation poses a significant challenge for components operating under frictional conditions, where surface degradation can lead to reduced performance and shortened service life.

Among the well-known methods are mechanical techniques, such as Ultrasonic Nanocrystalline Surface Modification (UNSM), which enable the formation of strain-hardened surface layers on aluminium alloy surfaces to enhance their mechanical properties [3,4]. Surface texturing, also classified as a mechanical technique, combined with using solid lubricants, has also significantly demonstrated the ability to reduce friction and wear under dry sliding conditions [5,6,7]. Chen et al. demonstrated microtexturing of aluminium alloy surfaces in combination with anodising and solid lubricants. [8]. In recent decades, significant research efforts have also been directed toward developing modifications that utilise anodising in combination with solid lubricants [9,10,11,12], metal–organic frameworks (MOFs) [13], or layered double hydroxide (LDH) conversion coatings [9,14,15] to protect metals against corrosion and/or enhance the tribological performance of anodic coatings formed on aluminum alloys. Anodising continues to be a widely used and easily scalable process in industrial applications, especially when compared to precision laser equipment, which may present greater challenges when applied to large surface areas or components with complex geometries.

The mechanical and tribological properties of the oxide coating produced during the anodising process are strongly influenced by the surface condition, porosity, and thickness of the Al_2_O_3_ layer. These, in turn, are highly dependent on the anodising conditions, such as voltage, temperature, and electrolyte composition [16,17,18]. The porosity of anodic oxide layers is considered a functional feature that is capable of acting as a reservoir for lubricants. This characteristic enables the design of self-lubricating systems that effectively reduce friction and wear [19,20,21].

Tribological wear tests of material pairs under dry (unlubricated) conditions are crucial for ensuring mechanical systems’ reliability, durability, and efficiency, especially in applications where lubricating oil is not feasible. Fundamental studies on oxide coatings formed on aluminium alloys support the selection of material pairs that exhibit the lowest wear rates and the highest durability under specific conditions. These investigations also help determine how long such coatings effectively protect the substrate and under what conditions they begin to degrade. Kim et al. [22] conducted tribological tests on nanoporous anodic aluminium oxide (AAO) coatings with varying pore sizes in sliding contact with a steel ball, under normal loads ranging from 1 mN to 1 N. The results showed that larger pore sizes corresponded to higher coefficients of friction. Additionally, it was observed that the coefficient of friction significantly decreased with increasing load for all AAO layers, regardless of their pore size. Hu et al. [23] conducted tribological tests on AAO coatings embedded with C60 nanoparticles, using a GCr15 bearing steel pin in reciprocating motion under dry friction conditions and a normal load of 5 N. They found that appropriate treatment to enlarge the nanopores of the AAO coating, combined with incorporating C60 particles, effectively reduced the coefficient of friction between the tested pair. Moreover, the modified AAO coating demonstrated good wear resistance. Lee et al. [24], in ball-on-disc sliding wear tests using a bearing steel ball and a PAA (porous anodic alumina)-coated disc, observed that water penetrating the pores is released onto the surface through elastic–plastic deformation. This released water acts as a lubricating film, which minimises direct contact between the surfaces and thereby reduces the coefficient of friction. In the study by Takaya et al. [25], friction measurements were carried out in a rotating ball-on-disc configuration, using a bearing steel ball and an AAO coating impregnated with PVP-iodine. It was found that the modified AAO coating exhibited a lower coefficient of friction and greater durability compared to the non-impregnated AAO coating. Maejima et al. [26] conducted friction measurements using a ring-on-disc (hardened steel ring-disc) and ball-on-disc (WC-Co ball-disc) configuration. The disc consisted of an AAO coating impregnated with molybdenum sulfide compounds. Both impregnated and non-impregnated samples were compared. It was found that the particle size of the solid lubricants was larger than the pore size of the AAO films, preventing direct impregnation of the lubricant powder into the pores. Nevertheless, it was confirmed that molybdenum sulfide and related compounds impregnated the AAO structure through the pore bases, significantly reducing friction and improving the running-in behaviour compared to the unmodified coating. Tao et al. [27] conducted friction and wear tests using a ball-on-disc tribometer, with a steel ball and an AAO disc modified with MoS_2_, under a load of 5 N. They found that MoS_2_ particles transferred during friction reduced the coefficient of friction until they were completely depleted. However, not all MoS_2_ particles present in the pores could be easily delivered to the contact interface. As a result, once the available MoS_2_ was exhausted, the friction between the AAO layer and the steel ball increased. The amount of MoS_2_ reaching the sliding interface was insufficient to form a continuous lubricating film between the surfaces. Nonetheless, it was confirmed that the MoS_2_ present within the pores of the oxide layer contributed to a reduction in the wear of the steel ball. Skeldon, Wang, and Thompson [10,28] found that a hard-anodic coating, combined with an incorporated solid lubricant like MoS_2_, MoS_3_, and S, can significantly enhance the tribological properties of aluminium compared to a conventional porous aluminium oxide layer. Their studies demonstrated that the introduced solid lubricant reduced the wear rate of aluminium in tribological contact with a steel pin.

Lubricated conditions significantly reduce friction and wear by forming a protective film between contacting surfaces. However, in many practical applications—such as in aerospace, automotive, or precision mechanical systems—lubrication may be limited, intermittent, or even undesirable due to design, environmental, or maintenance constraints. Materials and coatings must provide reliable tribological performance under dry conditions in such cases. Therefore, evaluating the effectiveness of IF-WS_2_-modified Al_2_O_3_ coatings in dry sliding is essential for understanding their suitability for these demanding environments, where conventional lubrication cannot be applied.

This work builds on our previous studies of the same coating system [29], where detailed structural and compositional analyses (including EDS) confirmed the successful incorporation and distribution of IF-WS_2_ nanoparticles; the current study focuses specifically on the tribological behaviour of the coating. The primary objective of this study is to investigate the influence of normal load and IF-WS_2_ modification on the wear behaviour of an Al_2_O_3_ coating applied to an EN-AW 5251 aluminium alloy in combination with ductile iron. The operating conditions modelled in this study simulate dry, reciprocating sliding contact between Al_2_O_3_ and Al_2_O_3_/IF-WS_2_ coatings and a ductile iron counter-body. These conditions represent tribological contacts found in lightweight mechanical systems, such as sliding elements in automotive, aerospace, and industrial equipment, where lubrication is limited or not feasible. The expected scientific contribution lies in determining whether IF-WS_2_ nanoparticles act as effective solid lubricants when embedded in Al_2_O_3_ coatings and whether this effect depends on the applied load. The worn surfaces were examined using scanning electron microscopy (SEM) and energy-dispersive spectroscopy (EDS). Based on statistical analysis, the results include a comprehensive evaluation of the coefficient of friction, wear intensity, and surface geometric parameters (SGPs). Due to the lack of available literature on this specific material combination (Al_2_O_3_/IF-WS_2_ coatings versus ductile iron pins), our findings provide novel data for solid lubrication and wear-resistant coatings. The paper uses statistical methods applied to DOE (Design of Experiments) design and analysis, which are helpful for process optimisation and finding relationships between input variables and measured parameters. DOE methodology is described in the studies [30,31,32]. However, to the authors’ knowledge, such a study has not yet been reported for tribological studies between cast iron pins and IF-WS_2_ modified oxide coatings on aluminium.

## 2. Materials and Methods

### 2.1. Sample Preparation

The investigated material was a composite oxide film consisting of Al_2_O_3_ and IF-WS_2_, fabricated through a two-step process. In the first stage, a graded Al_2_O_3_ coating was formed on the EN AW-5251 aluminium alloy via electrochemical oxidation in a ternary acid solution comprising sulfuric, oxalic, and phthalic acids (Avantor Performance Materials Poland S.A., Gliwice, Poland). Before anodisation, the samples underwent sequential etching in aqueous solutions of KOH and HNO_3_ (both from Avantor Performance Materials Poland S.A., Gliwice, Poland). Hard anodisation was carried out at a charge density of 180 A·min/dm^2^, under conditions of controlled current density, fixed duration, and a constant electrolyte temperature of 303 ± 1 K. In the second stage, IF-WS_2_ nanoparticles (NPs; NanoMaterials Ltd., Yavne, Israel) were introduced into the porous alumina layer. In Table 1, the sonication parameters are presented. The anodised specimens were immersed in room temperature ethanol containing 15 g/L of IF-WS_2_ NPs, subjected to ultrasonication at 20 kHz with a total energy input of 10 kJ for 5 min using a VCX 130 sonicator (Sonics & Materials Inc., Newtown, CT, USA), and subsequently left undisturbed for 24 h to facilitate nanoparticle incorporation [29].

### 2.2. Research Methods

To investigate the composition of nanoscale Al_2_O_3_ fibres incorporating self-organised IF-WS_2_ nanoparticles (NPs), the prepared samples were examined using a HITACHI S-4700 scanning electron microscope (SEM) (Hitachi High-Tech Corporation, Tokyo, Japan) equipped with a NORAN Vantage digital energy-dispersive X-ray spectroscopy (EDS) system (NORAN Instruments, Inc., Middleton, WI, USA). The surface and cross-sectional morphology of the Al_2_O_3_/IF-WS_2_ NP coatings was analysed in backscattered electron (BSE) mode using an yttrium aluminium garnet (YAG) detector (Hitachi High-Tech Corporation, Tokyo, Japan). A YAG single crystal was employed to reduce the electron dose and minimise beam-induced artefacts. Cross-sectional observations were performed on freshly fractured specimens. Before imaging, a thin carbon film was sputter-coated onto the sample surfaces to enhance contrast and reduce charging effects during SEM analysis. Microscopic observations of the ductile iron and Al_2_O_3_/IF-WS_2_ with wear track were conducted using a scanning electron microscope (SEM) with EDS JEOL 6480 (JEOL, Ltd., Tokyo, Japan). A Samsung Galaxy A32 mobile phone camera (Samsung Electronics, Suwon, Republic of Korea) was used for macroscopic imaging.

The coating thickness was measured using a contact method with the Fischer Dualoscope MP40 device (Helmut Fischer GmbH + Co. KG, Sindelfingen, Germany). The instrument operates based on the eddy current method. Ten measurements were performed on the surface of each sample, and the results were used to calculate the mean coating thickness along with the corresponding standard deviations.

Amplitude parameters, Abbott–Firestone bearing curves, and surface roughness profiles were determined based on profilometric measurements conducted before and after the tribological test. The measurements were performed using a Form TalySurf Series 2.50i contact profilometer (Taylor Hobson Ltd., Leicester, UK).

The samples prepared as described were subjected to tribological testing using a T-17 tribotester (Łukasiewicz Research Network—Institute for Sustainable Technologies, Radom, Poland). The tests were conducted in a pin-on-plate configuration under reciprocating motion at room temperature and a relative humidity of 40% ± 10%. An average sliding speed of 0.2 m/s was maintained, corresponding to an operating frequency of 2.5 Hz. All tests were performed under dry friction conditions. Tribological tests were conducted under loads of 0.3 MPa (~19N) and 0.6 MPa (~38N). The total sliding distance for each tribological pair was 1 km. A ductile iron pin with a diameter of 9 mm was employed as the counter-sample. The tests yielded frictional characteristics, including friction force data. The average coefficient of friction was calculated based on the stabilised regions of the friction force profile. Before each dry friction test, the ductile iron pin was lapped using P1000-grade sandpaper (Union Abrasives, Inc., Pepperell, MA, USA) for 10 min under a constant load of 3.242 kg. This procedure ensured uniform surface roughness of the counter-sample, thereby standardising the initial conditions across all test runs. The wear of the ductile iron pin was evaluated by measuring its mass before and after each test cycle using a WPA-60G analytical balance (Radwag, Radom, Poland) with a precision of ±0.1 mg.

A 2^k^ factorial design experiment with replication was conducted using Statistica v. 13 software (TIBCO Software Inc., Palo Alto, CA, USA) to evaluate the influence of input parameters on the dependent variables (Table 2). The independent variables were those doped with IF-WS_2_ or undoped Al_2_O_3_ coating and load (0.3 or 0.6 MPa). The dependent variables for wear resistance properties were structural geometric parameters (SGPs), friction coefficient μ, and the intensity of pin wear.

## 3. Results and Discussion

**Figure 1:**
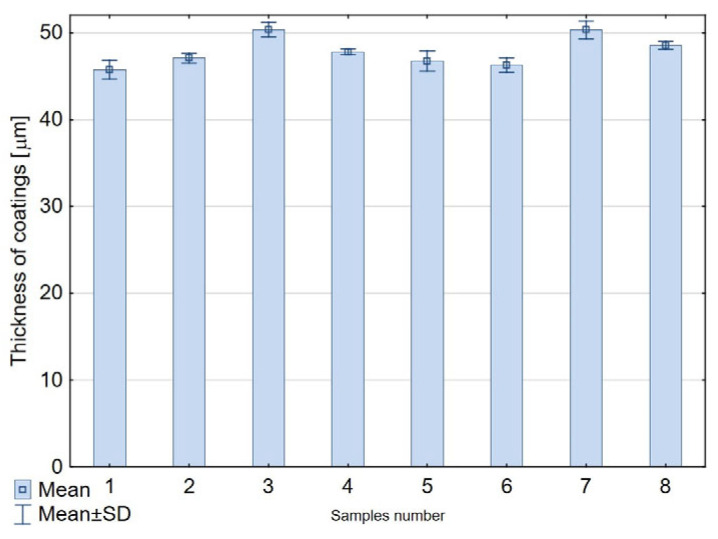
Thickness of coatings.

**Figure 2:**
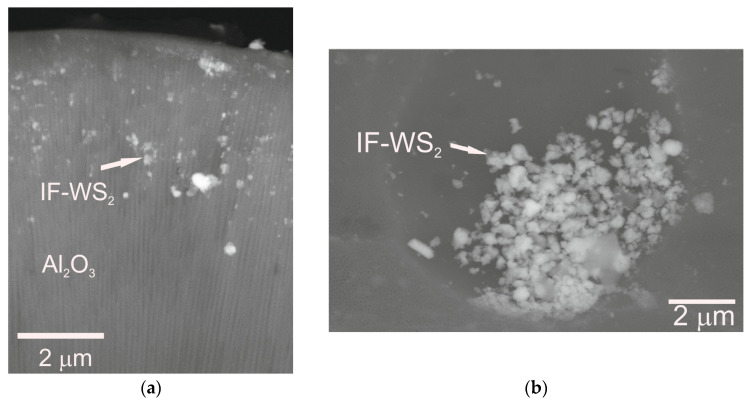
Microstructures of (**a**) the fresh cross-section (×10,000) and (**b**) the surface of the Al_2_O_3_/IF-WS_2_ coating.

As a result of the electrochemical process, oxide coatings with the thicknesses presented in Figure 1 were obtained. The application of ultrasound in a sonicator enabled the deagglomeration of the IF-WS_2_ nanopowder and, in combination with a 24 h sedimentation method, facilitated its incorporation into the nanoporous structure of the Al_2_O_3_ coating (Figure 2). Photo 2a shows the IF-WS_2_ nanopowder introduced between Al_2_O_3_ nanofibers on the coating of the fresh fracture. Photo 2b shows the IF-WS_2_ nanopowder nested in the macropores on the Al_2_O_3_ coating surface.

**Figure 3:**
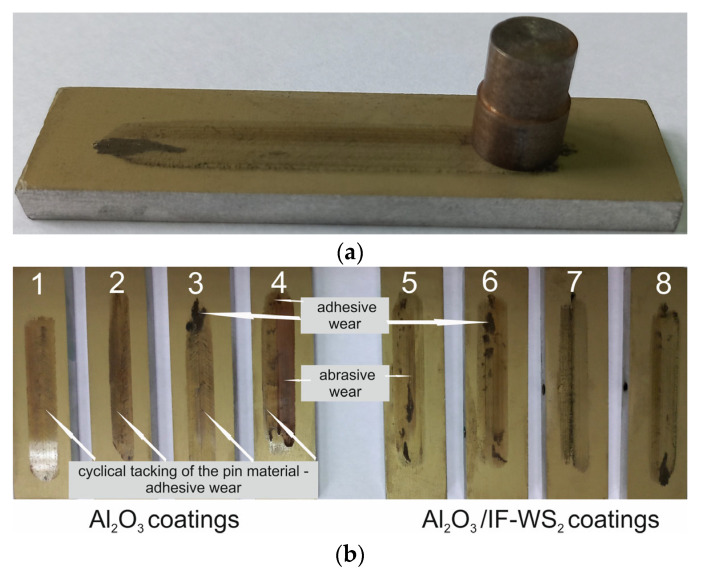
(**a**) An example of a friction pair: oxide coating–cast iron pin; (**b**) photos of friction traces on unmodified Al_2_O_3_ oxide coatings (1–4) and modified Al_2_O_3_/IF-WS_2_ (5–8).

Figure 3a shows an example of a friction pair: oxide coating–cast iron pin. Figure 3b shows a photo of the oxide coating with a visible wear track resulting from interaction with a cast iron pin. Friction traces on unmodified Al_2_O_3_ oxide coatings are shown on samples 1–4, and modified Al_2_O_3_/IF-WS_2_ oxide coatings are shown on samples 5–8. Figure 3b shows a noticeable difference in the deposited cast iron film between the unmodified and modified samples. The transfer film on the modified Al_2_O_3_/IF-WS_2_ coatings appears thinner overall (adhesive wear), with more frequent occurrences of isolated, thicker regions of deposited cast iron (abrasive wear) than the unmodified Al_2_O_3_ coatings. In the case of unmodified Al_2_O_3_ coatings, in addition to the friction traces, a cycling tacking of the pin material (parallel marks) was observed on the coating surface, which may correspond to coating cracks. Such traces were not observed on the modified Al_2_O_3_/IF-WS_2_ coating surface. Similar coating distortions were observed in [33], where coatings without cracks were more wear resistant. What should be emphasised, however, regardless of whether the Al_2_O_3_ coatings were modified or not, the coatings were not broken after friction, and a well-adhering sliding film formed from the wear products of the cast iron pin remained on their surface. The adhesive bonds observed in some cases in the tribofilm are richer in iron, which suggests a thicker sliding film in these places. In the unmodified Al_2_O_3_ coatings, repetitive parallel marks were observed after the tribological interaction, which are indicative of adhesive wear from the ductile iron counter-body and are likely associated with microcracks in the Al_2_O_3_ coatings. These marks were not observed in the Al_2_O_3_/IF-WS_2_ coatings, suggesting a change in the wear mechanism. The presence of IF-WS_2_ nanoparticles, known for their solid lubrication properties, likely reduced the coefficient of friction and localised stresses at the contact interface. This, in turn, minimised the extent of parallel adhesive interactions and prevented crack formation.

**Figure 4:**
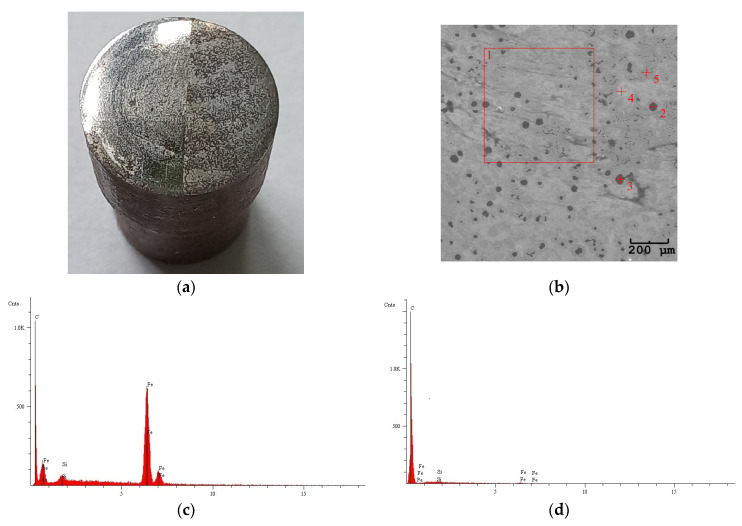

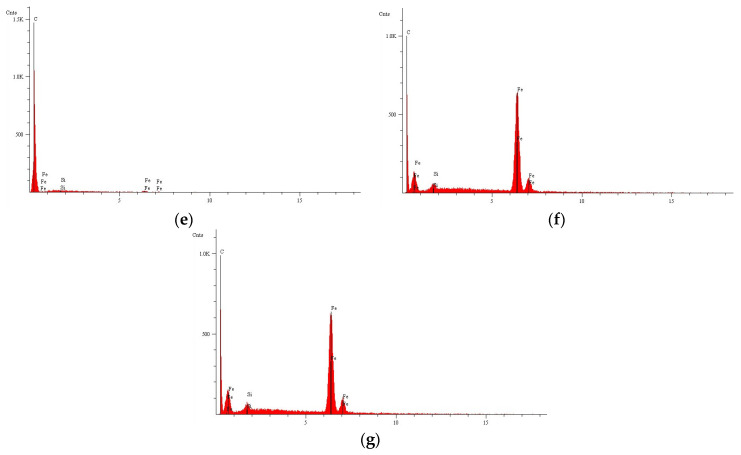
(**a**) Picture of the cast iron pin; (**b**) SEM image of the etched surface; (**c**) EDS analysis of area 1 on the SEM image; (**d**) EDS analysis of point 2 on the SEM image; (**e**) EDS analysis of point 3 on the SEM image; (**f**) EDS analysis of point 4 on the SEM image; (**g**) EDS analysis of point 5 on the SEM image.

Figure 4 shows an image of the cast iron pin used in the friction tests, accompanied by SEM images of the etched surface and EDS analysis of the selected areas.

**Figure 5:**
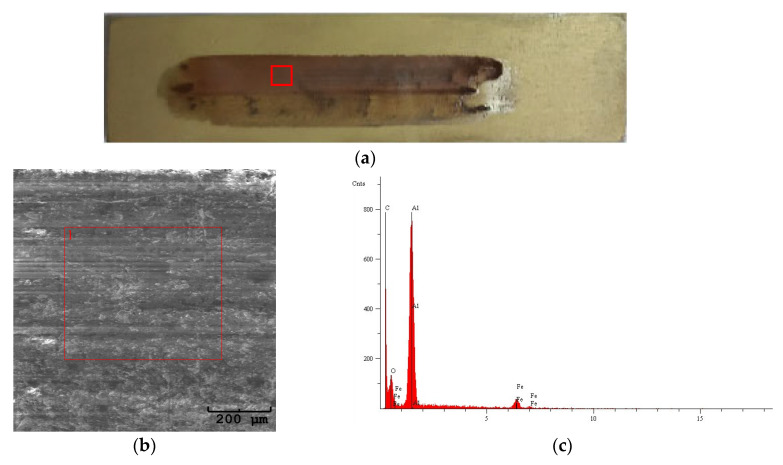
(**a**) Wear track on sample 4, non-modified Al_2_O_3_; (**b**) SEM image of the wear track; (**c**) EDS analysis of area 1 on the SEM image.

**Figure 6:**
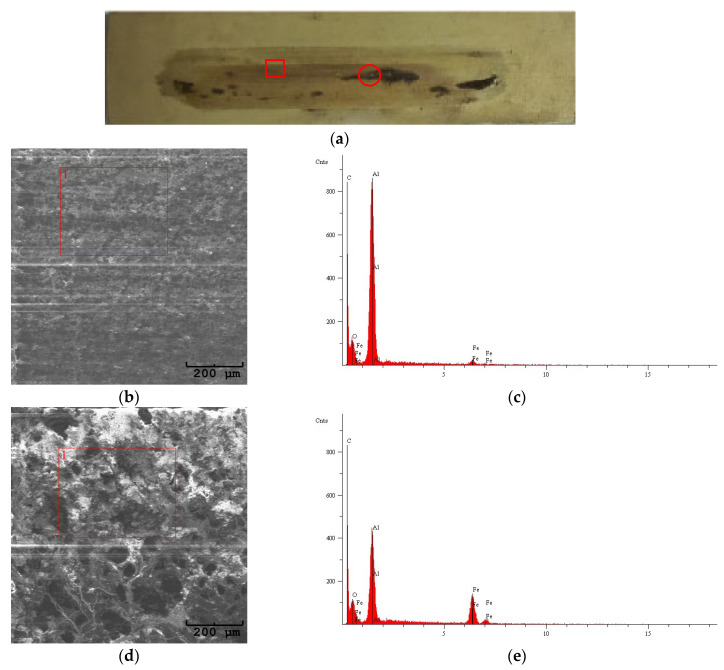
(**a**) Wear track on sample 5, modified Al_2_O_3_/IF-WS_2_; (**b**) SEM image of the wear track from the rectangular area in the “**a**” image; (**c**) EDS of area 1 on the “**b**” SEM image; (**d**) SEM image of the wear track from the circular area in the “**a**” image; (**e**) EDS of area 1 on the “**d**” SEM image.

Figure 5 and Figure 6 show images of the wear tracks, SEM images of the wear tracks, and EDS analysis of the selected areas. As observed on both the unmodified Al_2_O_3_ and modified Al_2_O_3_/IF-WS_2_ coatings, a transfer film from the counter-face was deposited (Figure 5 and Figure 6a), which was identified as spheroidal cast iron through EDS analysis (Figure 5c and Figure 6c,e). In areas where thicker deposits were visible on the Al_2_O_3_ coatings, increased intensity of the iron reflection characteristic of the spheroidal cast iron was recorded, confirming the presence of a thicker transfer film at these locations.

**Figure 7:**
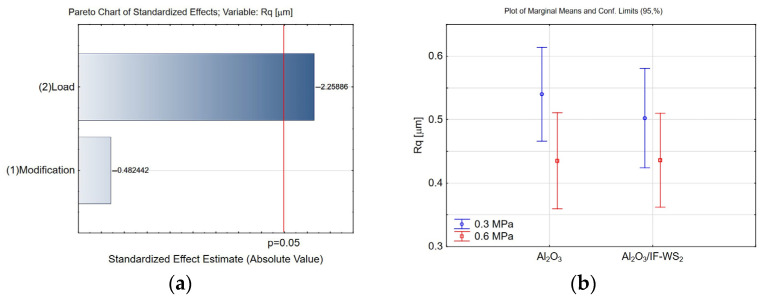
(**a**) Pareto chart of the standardised effect for the Rq parameter; (**b**) plot of marginal means and 95% confidence limits for the Rq parameter.

**Figure 8:**
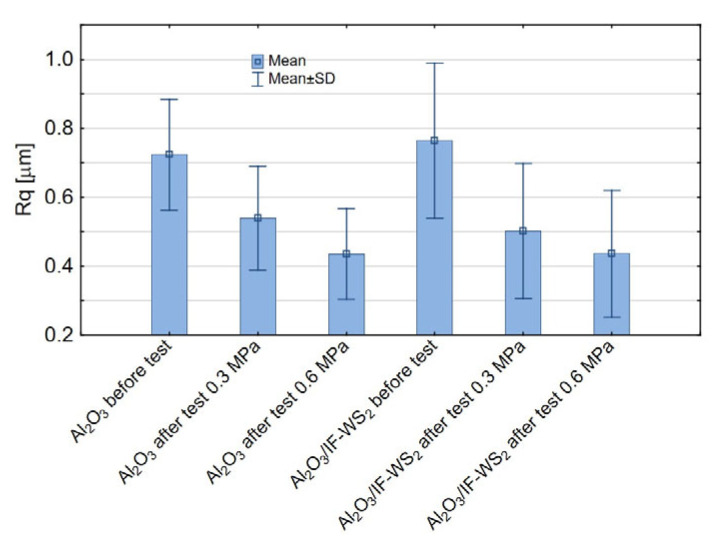
Means and standard deviation for the Rq parameter.

The following parameters were selected to analyse the influence of the additive and applied load on surface geometric parameters (SGP): Rq, Rk, Rvk, Rpk, and Rsk. The Rq parameter, representing the root mean square deviation of the surface profile, is particularly sensitive to larger surface irregularities. It is commonly used in tribological studies and recommended when it is essential to accounting for peaks and valleys in greater detail. Surface roughness significantly affects the contact area between interacting surfaces—higher Rq values indicate a rougher surface, which may increase friction or alter the wear mechanism. Roughness also plays a role in lubricant retention—microvalleys can trap lubricant, thereby enhancing tribological performance. Conversely, depending on material type and operating conditions, lower Rq values may lead to reduced wear depending on material type and operating conditions. In nanolubricant deposition, surface valleys are significant in the present study. Pareto and marginal mean charts were used to analyse the results. The most commonly used significance level of 0.05 (5%) was used in the presented studies. The significance level and confidence intervals are closely related by the relationship (1 − α = 0.95). This means that there is a 95% chance that the confidence interval will include the actual values of the parameter. In Pareto charts, the horizontal axis shows the normalised effect values (absolute values), while the vertical line indicates the statistical significance threshold at *p* = 0.05. Factors on this line’s right side are considered statistically significant. As shown in Figure 7a, within the adopted experimental design, only the applied load significantly influenced the Rq parameter. Specifically, lower Rq values were observed under higher load conditions (0.6 MPa), as illustrated in Figure 7b. This suggests that roughness is sensitive to mechanical loading, reflecting intensified wear. Based on Figure 8, and considering the measurement uncertainty, it can be assumed that the initial surface roughness Rq of both unmodified Al_2_O_3_ and modified Al_2_O_3_/IF-WS_2_ coatings was similar, approximately 0.7 µm before the tribological tests and 0.5 µm (0.3 MPa load) and 0.43 µm (0.6 MPa load) after the tests. The decrease in Rq observed after the tribological tests indicates a typical smoothing of the friction surfaces, resulting from deformation and partial shearing of the Al_2_O_3_ coating asperities, as well as the filling of surface valleys with wear debris originating from cast iron. An increase in load intensifies these mechanisms—higher contact pressure leads to deeper deformation and greater removal of material from surface asperities, which results in a more pronounced reduction in the Rq parameter. The absence of significant changes in Rq due to IF-WS_2_ modification indicates that the additive did not strongly affect the surface topography in terms of measurable roughness, despite influencing the friction behaviour.

The Rk parameter, derived from the Abbott–Firestone bearing curve (also known as the material ratio curve), belongs—along with Rpk and Rvk—to the roughness parameters defined in ISO 21920-2 [34]. These parameters provide a more comprehensive interpretation of the microtopography of a surface. Rk represents the core roughness depth, corresponding to the portion of the surface profile that primarily carries the load during contact—it reflects the main load-bearing capacity of the surface.

**Figure 9:**
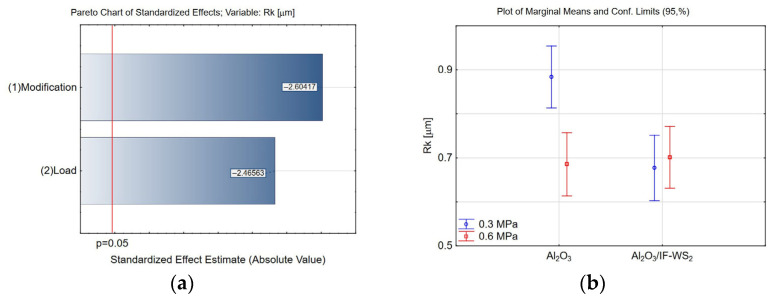
(**a**) Pareto chart of the standardised effect for the Rk parameter; (**b**) plot of marginal means and 95% confidence limits for the Rk parameter.

**Figure 10:**
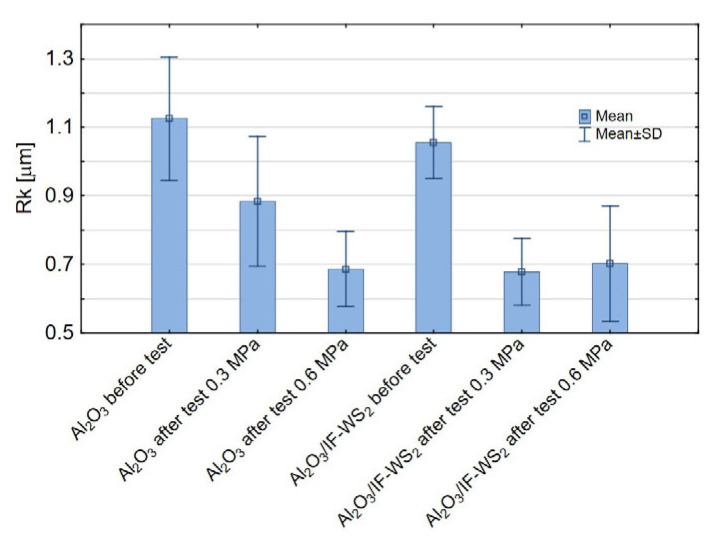
Means and standard deviation for the Rk parameter.

Analysis of the two-level factorial experimental design revealed that the Rk parameter is significantly influenced by both surface modification and applied load (Figure 9a). At a load of 0.3 MPa, a reduction of approximately 23% in the Rk value was observed for the modified Al_2_O_3_/IF-WS_2_ coating compared to the unmodified Al_2_O_3_ coating (Figure 9b). Additionally, compared with the initial (pre-test) Rk values, a decrease in Rk was noted for both the unmodified and modified coatings after the tribological tests (Figure 10). Modification did not significantly affect the Rk parameter under the higher load condition (0.6 MPa). Incorporating the IF-WS_2_ nanomodifier into Al_2_O_3_ coatings positively affects the load-bearing zone by reducing the Rk value, which may enhance the tribological durability of the tested material pairings under 0.3 MPa load. However, at 0.6 MPa, modification appears to have no significant influence.

**Figure 11:**
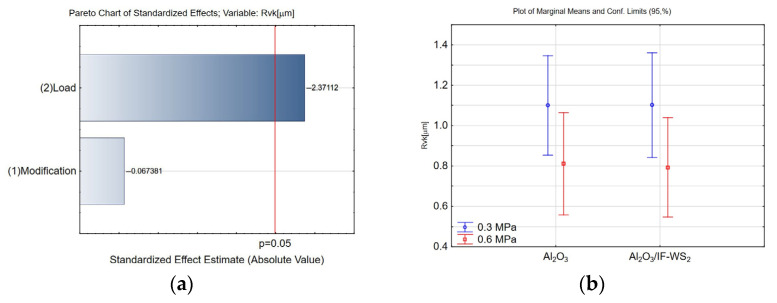
(**a**) Pareto chart of the standardised effect for the Rvk parameter; (**b**) plot of marginal means and 95% confidence limits for the Rvk parameter.

The Rvk parameter defines the depth of valleys capable of retaining lubricant. According to the statistical analysis, Rvk is influenced solely by the applied load during the tribological test (Figure 11a). At a lower load of 0.3 MPa (Figure 11b), more surface valleys are preserved—these features are inherent to the structure of the anodised coating. As the applied load increases to 0.6 MPa, more valleys are lost—the surface becomes flattened and more uniform. This effect results from the mechanical flattening of the Al_2_O_3_ coating surface under the load applied by the pin and the filling or smoothing of surface valleys with material from the pin itself, which in this study was made of spheroidal graphite cast iron.

**Figure 12:**
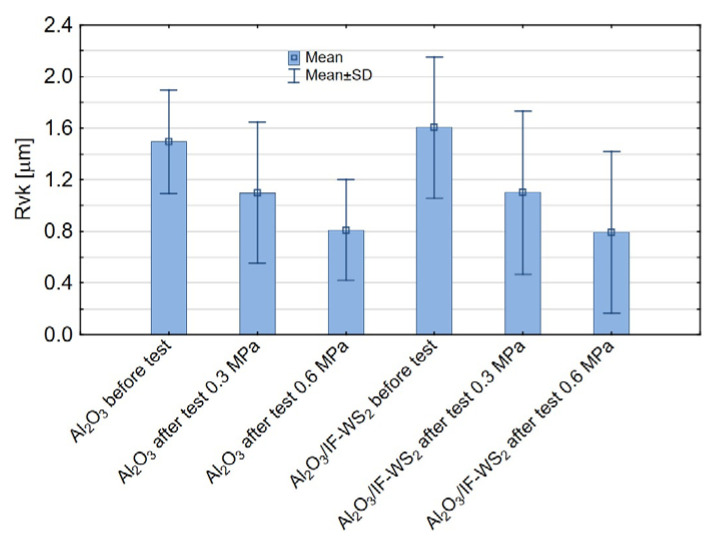
Means and standard deviation for the Rvk parameter.

As shown in Figure 12, the Rvk parameter decreases after the tribological tests compared to the surface condition before testing.

**Figure 13:**
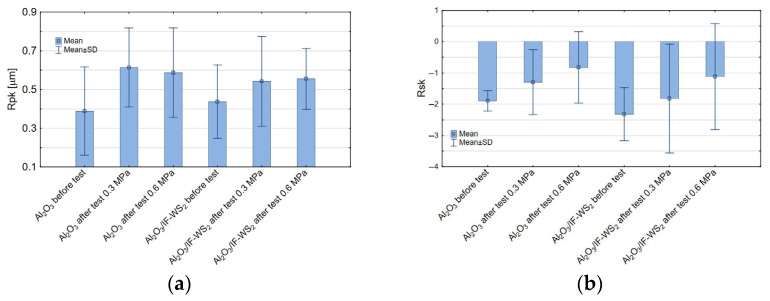
Means and standard deviation for (**a**) the Rpk parameter and (**b**) the Rsk parameter.

The third parameter derived from the Abbott–Firestone bearing curve, Rpk, was unaffected by either surface modification or the applied load. Therefore, Figure 13a presents only the changes in this parameter following the tribological tests, compared to the pre-test values. In both cases, Rpk increased after testing. Rpk characterises the easily removable surface peaks that typically wear off during the initial stages of contact. It is generally expected to decrease after tribological interaction. However, in this study, an increase was observed. This phenomenon is attributed to the formation of a transferred cast iron sliding film, which protrudes above the functional surface—these are adhesive junctions or transfer layers resulting from material transfer from the cast iron pin. Similar observations were made for the Rsk parameter, which describes the asymmetry (skewness) of the surface profile and was likewise found to be independent of the input variables examined (Figure 13b). A negative Rsk value indicates a surface dominated by valleys, which can enhance lubricant retention and reduce friction but may also reduce the load-bearing capacity. Based on average values (without accounting for standard deviation), it was noted that increasing the load raised the skewness coefficient, thereby reducing the surface’s capacity to retain lubricant.

**Figure 14:**
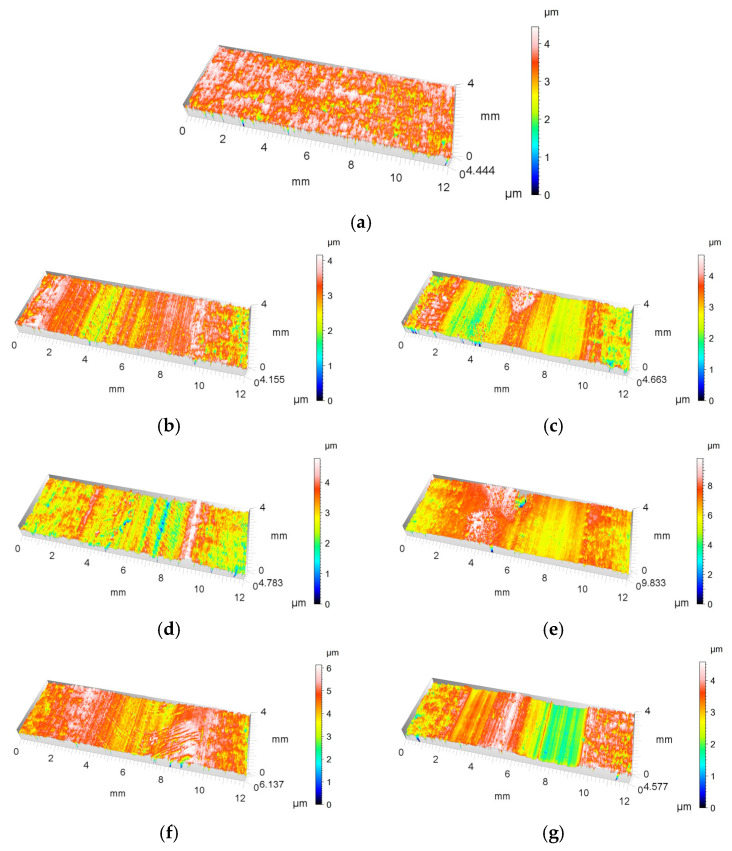

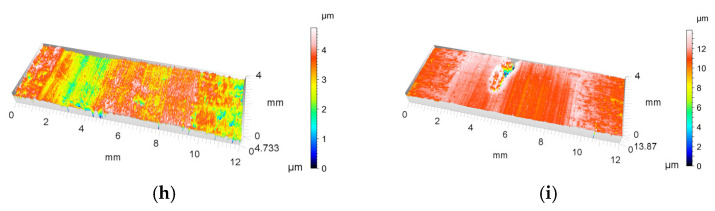
3D visualisation of (**a**) the surface of Al_2_O_3_ before tribological test; (**b**,**d**,**f**,**h**) the unmodified Al_2_O_3_ surface after the tribological test for samples 1, 2, 3, and 4, respectively; (**c**,**e**,**g**,**i**) the modified Al_2_O_3_/IF-WS_2_ surface after the tribological test for samples 5, 6, 7, and 8, respectively.

Figure 14 presents a 3D visualisation of the Al_2_O_3_ oxide coatings: Figure 14a shows the coating surface before the tribological test, and Figure 14b,d,f,h depict the unmodified Al_2_O_3_ coatings after testing for samples 1, 2, 3, and 4, respectively. Figure 14c,e,g,i illustrate the modified Al_2_O_3_/IF-WS_2_ coatings after testing for samples 5, 6, 7, and 8, respectively. The surface before friction (Figure 14a) is characterised by a uniform topography with minor height differences, mainly in the 1–2.5 µm range, locally up to 4 µm. The natural roughness results from the coating manufacturing process. In each case of the 3D image shown, parallel bands characteristic of sliding contact resulting from abrasive wear are visible. The significantly increased intensity of the yellow, green, and red colours suggests local deformations and microcracks. The material peaks marked in white suggest cast iron material transfer (adhesion). After friction, all surfaces show a slight degree of wear, with small surface deformation. Observation of the 3D surface images revealed that the surface profile of the Al_2_O_3_ coatings—both unmodified and modified—exhibited only minor deformation in the texture as a result of interaction with the cast iron pin.

**Figure 15:**
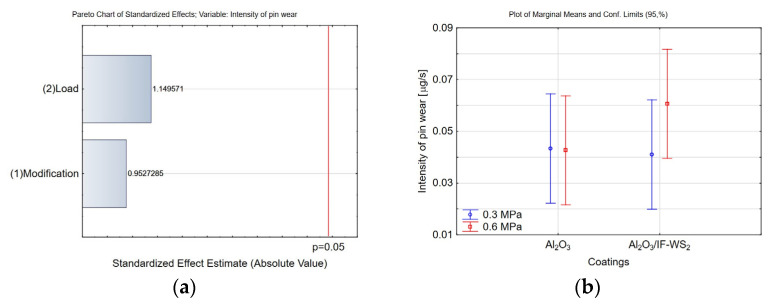
(**a**) Pareto chart of the standardised effect for the intensity of the wear pin; (**b**) plot of marginal means and 95% confidence limits for the intensity of the wear pin.

As shown in the Pareto and marginal means charts (Figure 15a,b, respectively), neither the surface modification nor the applied load had a statistically significant effect on the wear intensity of the cast iron pin.

**Figure 16:**
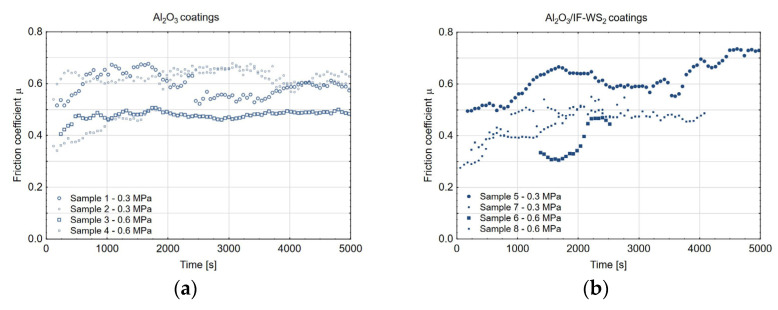
Friction coefficient vs. time for (**a**) the Al_2_O_3_ coatings and (**b**) the Al_2_O_3_/IF-WS_2_ coatings.

**Figure 17:**
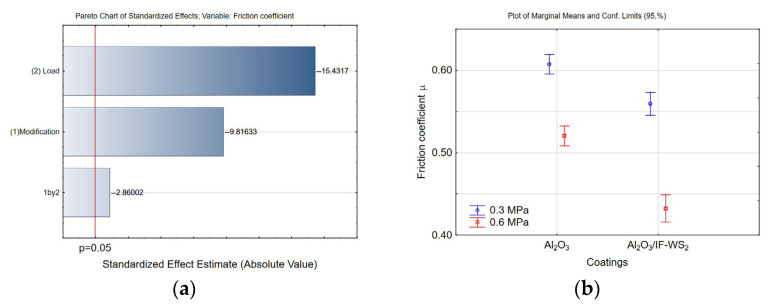
**(a**) Pareto chart of the standardised effect for the friction coefficient μ; (**b**) plot of marginal means and 95% confidence limits for the friction coefficient μ.

Figure 16 illustrates the friction coefficient as a function of time for all tested samples. According to the Pareto chart (Figure 17a), both the applied load and the surface modification had a statistically significant impact on the friction coefficient, with the load having a more pronounced effect. As shown in Figure 17b, the friction coefficient was lower for Al_2_O_3_ coatings doped with the IF-WS_2_ nanolubricant, by approximately 9% at a load of 0.3 MPa and 15% at a load of 0.6 MPa. It can be hypothesised that at the lower load of 0.3 MPa, a greater number of valleys—originating from the anodised coating structure—remain intact (see Figure 11b), which limits the interaction of the loosely embedded IF-WS_2_ nanolubricant with the load-bearing surface area. In contrast, under higher load conditions, the lubricating effect of the additive is enhanced due to the disruption of Van der Waals forces. The fullerene-like structure of IF-WS_2_ breaks down, forming 2H-WS_2_ lamellae, which facilitate sliding. Additionally, the cast iron transfer film becomes enriched with these 2H-WS_2_ planes, further contributing to the observed reduction in the friction coefficient.

Although a reduction in friction coefficient is often expected to correspond with lower wear, this relationship is not universal—wear is governed by many factors: hardness, microstructural integrity, the presence of tribochemical layers, and rough surface features. According to Zhou et al. [35], hardness often plays a more significant role in determining wear rate than friction coefficient, especially in PVD coatings. As stated by Krella [36], a decrease in the coefficient of friction is often associated with a reduction in wear rate; however, no consistent correlation between these two parameters has been established. Moreover, some studies have reported cases where an increase in the coefficient of friction coincided with a reduction in wear rate, indicating that a low friction coefficient does not necessarily imply improved wear resistance. In our case, the applied IF-WS_2_ modification altered frictional behaviour—likely through changes in surface interactions or tribofilm formation—but did not significantly affect material properties such as hardness, structural integrity, or tribochemical stability. WS_2_ belongs to the transition metal dichalcogenides, part of a large class of so-called two-dimensional or layered materials. Within the layers, the atoms are bonded by strong covalent forces, while van der Waals forces hold together the individual layers. WS_2_, due to its layered structure and the weak van der Waals interactions between the layers, causes a reduction in the coefficient of friction through a peeling mechanism [37]. In the case of the fullerene-like IF-WS_2_ consisting of dozens of cage-like concentric layers [38], loading causes exfoliation of the outer layers of the nanoparticle, inducing slippage and a reduction in the coefficient of friction. Our results indicated that the addition of IF-WS_2_ does not influence the morphology of the coating, thereby not affecting its hardness and wear resistance. Furthermore, the addition of IF-WS_2_ does not affect the materials’ properties, such as hardness, the structural integrity of Al_2_O_3_ nanofibers, or tribochemical stability. Therefore, the absence of wear rate change is consistent with known tribological principles whereby these dominant factors more strongly control wear resistance than friction alone.

The present study demonstrates that modifying Al_2_O_3_ coatings with IF/WS_2_ nanopowder led to a moderate improvement in frictional behaviour when tested against a ductile iron counter-body. This limited enhancement may be attributed to the likely non-uniform distribution of IF/WS_2_ nanoparticles within the ceramic matrix. This aspect warrants further investigation and optimisation of the deposition process. Although ductile iron is not typically used in tribological studies involving Al_2_O_3_ coatings, its selection was intentional, aiming to replicate specific industrial conditions characterised by dry sliding contacts in lightweight mechanical systems. The adhesive wear mechanisms observed on the cast iron surface support the relevance of this material pairing. Future research should explore their tribological performance under varying environmental conditions, including elevated humidity and temperature fluctuations, which more accurately reflect real-world operational environments, to assess the functional potential of such coatings fully.

## 4. Conclusions

To investigate the potential application of IF-WS_2_ nanoparticles (NPs) in Al_2_O_3_ coatings for tribological studies involving spheroidal graphite cast iron, an Al_2_O_3_ coating embedded with IF-WS_2_ nanoparticles was fabricated using a sonication-assisted sedimentation technique. The effectiveness of this approach was evaluated through SEM analysis, surface geometric parameter (SGP) measurements, friction coefficient monitoring, and cast iron pin wear assessment. The main conclusions drawn from the study are as follows:

- SEM/EDS analysis confirmed the transfer of pin material onto the fabricated Al_2_O_3_ coatings. In some cases, adhesive junctions were observed, with localised accumulations of transferred spheroidal graphite iron.
- Three-dimensional surface imaging revealed that the unmodified and IF-WS_2_-modified Al_2_O_3_ coatings exhibited minor subsurface deformation due to sliding contact with the cast iron pin.
- In the analysed test setup, the applied load significantly influenced the Rq roughness parameter, while the IF-WS_2_ nanomodifier did not considerably affect surface roughness. This suggests that load conditions are more critical than material modification in the context of improving roughness (and potentially friction).
- The two-level factorial analysis demonstrated that modification and load significantly influenced the Rk load-bearing parameter. Incorporating the nanomodifier and increased load enhanced the load-bearing capacity of the surface by reducing the Rk value, thereby improving the tribological durability of the tested pairs.
- The Rvk parameter, related to valley depth for lubricant retention, depended solely on the load—lower loads helped preserve valleys within the coating structure. In contrast, higher loads led to valley reduction due to surface flattening and filling with pin material.
- Parameters Rpk (reduced peak height) and Rsk (profile skewness) showed no significant dependency on the input variables. However, higher loads increased Rpk, and the negative Rsk values indicated a valley-dominant surface morphology, which can enhance lubricant retention and reduce friction, albeit potentially at the expense of load-bearing capacity.
- Neither modification nor load significantly influenced the wear intensity of the cast iron pin.
- Both load and modification had a notable effect on the friction coefficient. The use of IF-WS_2_-modified Al_2_O_3_ coatings reduced the friction coefficient, and the coefficient also decreased with increasing load, by approximately 9% at a load of 0.3 MPa and 15% at a load of 0.6 MPa, suggesting enhanced lubricating performance under higher contact stress.

## Figures and Tables

**Table 1:** Sonication parameters.

Dispersion Liquid	Time [min]	Power [kJ]	Frequency [kHz]
Ethanol +15 g/L of IF-WS_2_ NPs	5	10	20

**Table 2:** Research test conditions.

Sample Designation	Test Input Variables	
	Introducing IF-WS_2_ NPs	Load [MPa]
1	No (−1)	0.3 (−1)
2	No (−1)	0.3 (−1)
3	No (−1)	0.6 (1)
4	No (−1)	0.6 (1)
5	Yes (1)	0.3 (−1)
6	Yes (1)	0.6 (1)
7	Yes (1)	0.3 (−1)
8	Yes (1)	0.6 (−1)

## Data Availability

The original contributions presented in this study are included in the article. Further inquiries can be directed to the corresponding authors.
